# Role and mechanism of hexyl-aminolevulinate ethosome-mediated antimicrobial photodynamic therapy in reversing fluconazole resistance in Nakaseomyces glabrata (Candida glabrata)

**DOI:** 10.1099/jmm.0.002060

**Published:** 2025-09-12

**Authors:** Yingzhe Wang, Yingting Huang, Wei Long, Shigan Ye, Yuan Deng, Sanquan Zhang, Huanli Wang, Xiaoliang Zhu

**Affiliations:** 1 Department of Dermatology, Nanfang Hospital, Southern Medical University, Guangzhou, PR China; 2 Department of Dermatology, Guangzhou Hospital of Integrated Traditional and Western Medicine, Guangzhou, PR China; 3 Guangzhou Dermatology Hospital, Guangzhou, PR China; 4 Institute of Dermatology, Guangzhou Medical University, Guangzhou, PR China

**Keywords:** azole resistance, efflux pump, HAL-ES-aPDT, *Nakaseomyces glabrata*

## Abstract

**Introduction.** Azoles are extensively employed as clinical antifungal agents; however, their long-term and widespread application contributed to the progressive emergence of azole resistance. A significant increase in infections caused by azole-resistant *Nakaseomyces glabrata* highlights the need for novel therapeutic strategies.

**Hypothesis/Gap Statement.** The efficacy of hexyl-aminolevulinate ethosome-mediated antimicrobial photodynamic therapy (HAL-ES-aPDT) against drug-resistant fungal pathogens, as well as its underlying mechanisms, remains to be elucidated. Our previous studies have demonstrated that HAL-ES-aPDT, which utilizes the photosensitizer HAL-ES, eliminates pathogens via photochemical reactions.

**Aim.** This study aimed to evaluate the effects and mechanisms of HAL-ES-aPDT on clinical isolates of *N. glabrata* exhibiting varying levels of azole susceptibility, with a focus on changes in resistance and virulence.

**Methodology.** Several clinical isolates of *N. glabrata* were collected, and the effects and mechanisms of HAL-ES-aPDT treatment on azole-resistant strains were investigated.

**Results.** HAL-ES-aPDT reduced *N. glabrata* tolerance to environmental stress and reversed azole resistance by inhibiting drug efflux and downregulating genes encoding the target enzymes. It also attenuated *in vivo* virulence by downregulating the expression of the epithelial adhesin gene *EPA1*.

**Conclusion.** These results confirm the efficacy of HAL-ES-aPDT against azole-resistant *N. glabrata* and provide novel mechanistic insights that may facilitate the development of effective therapeutic interventions for resistant fungal infections.

## Introduction

Over the past three decades, the incidence of infections caused by non-*Candida albicans* species has significantly increased [1]. Among these, *Nakaseomyces glabrata* is of particular clinical relevance [1]. Formerly classified as *Candida glabrata*, it was reclassified in 2022 following phylogenetic and genomic analyses that revealed considerable similarities with species in the *Nakaseomyces* genus, including conserved gene organization, gene synteny and specific gene family profiles [2,3]. The prevalence of *N. glabrata* infections has increased across various regions, accounting for 7.4%–21.1% of clinical *Candida* isolates, and is currently recognized as the second most common causative agent of candidiasis [3,4]. Similar to other *Candida* species, *N. glabrata* is a constituent of the human commensal microbiota and is capable of colonizing multiple anatomical sites, including the skin, oral cavity, gastrointestinal tract and urinary system [5]. Azoles are the only class of antifungal agents used for the long-term oral treatment of deep-seated fungal infections. Owing to their high oral bioavailability, broad-spectrum antifungal activity and favourable safety profiles, they are widely utilized in clinical practice [6]. However, *N. glabrata* exhibits intrinsic genetic resistance to azole antifungal agents and possesses a pronounced capacity for developing acquired resistance [7,8]. Epidemiological data indicate that 62.8%–76.7% of *N. glabrata* clinical isolates exhibit decreased susceptibility to fluconazole, with 8.1%–16.5% demonstrating confirmed azole resistance [4,9].

Antimicrobial photodynamic therapy (aPDT) is recognized for its effectiveness against infections caused by difficult-to-treat pathogens [10]. It operates by utilizing photosensitizers that, upon laser irradiation, generate reactive oxygen species (ROS) capable of damaging intracellular biomolecules. Owing to its physical mode of action and multi-targeted oxidative stress, aPDT does not promote the induction of pathogen resistance [11,12]. However, the clinical applicability of aPDT remains limited by the availability of highly effective photosensitizers. To address this limitation, a novel antibacterial photosensitizer, hexyl-aminolevulinate ethosomes, was developed by combining the high lipophilicity of hexyl-aminolevulinate (HAL) with the superior stability and transdermal permeability of an ethosome. This formulation enables deeper tissue penetration and efficient production of protoporphyrin IX. Compared with the classical photosensitizer 5-aminolevulinic acid, hexyl-aminolevulinate ethosome (HAL-ES) exhibits improved permeability and photosensitivity, supporting its potential as a safe, efficient and cost-effective therapeutic agent [13,14]. In our previous studies, hexyl-aminolevulinate ethosome-mediated antimicrobial photodynamic therapy (HAL-ES-aPDT) demonstrated potent antimicrobial activity against *Propionibacterium acnes*, *Staphylococcus aureus* and *C. albicans* [15,17].

Although HAL-ES-aPDT has shown broad-spectrum antimicrobial activity, its therapeutic efficacy and underlying mechanism against *N. glabrata* remain to be elucidated. In this study, HAL-ES-aPDT was applied to clinically isolated azole-resistant *N. glabrata* strains to investigate its mechanism of action. The findings aim to support the development of an effective therapeutic strategy for treating azole-resistant *N. glabrata* infections.

## Methods

### Strains, growth conditions, and reagents

The reference strain *N. glabrata* ATCC 2001 was purchased from the Guangdong Microbial Culture Collection Center. Clinical isolates of *N. glabrata* were collected from the Department of Laboratory Medicine, Nanfang Hospital, Southern Medical University (Table S1, available in the online Supplementary Material). Informed consent was obtained from patients’ families. Species identification was performed by automatic mass spectrometry using Matrix-Assisted Laser Desorption/Ionization Time of Flight (MALDI-TOF) technology [18,19]. After identification, ChromAgar plates were used to confirm the result [20]. Antifungal susceptibility to azoles was initially assessed using the ATB Fungus Kit (Nanfang Hospital, Guangzhou, China). Fluconazole susceptibility was further confirmed by broth microdilution according to the M27-A3 guidelines recommended by the National Committee for Clinical Laboratory Standards. Based on fluconazole MICs, clinical isolates were categorized into three groups: susceptible (S1–S3, MIC ≤8 µg·ml^−1^), dose-dependent susceptible (I1–I3, MIC 8–64 µg·ml^−1^) and resistant (R1–R3, MIC ≥64 µg·ml^−1^).

All *N. glabrata* strains were cultured on Yeast Extract Peptone Dextrose (YPD) medium agar plates (0.5% yeast extract, 1% Bacto-Peptone, 2% glucose and 1.4% agar) and incubated at 37 °C. HAL-ES, containing 30% ES and 20 mM (5 mg ml^−1^) HAL (NMT Biotech, Suzhou, China), was prepared as previously described [15].

### Spot assay

A total of 39.2 g of YPD agar medium powder was weighed using an electronic analytical balance and dissolved in 800 ml of double-distilled water with continuous stirring. The solution was sterilized by autoclaving at 121 °C for 15 min. After cooling to an appropriate temperature, separate YPD media containing hydrogen peroxide (H₂O₂), 10 mM; SDS, 0.01%; and sodium chloride (NaCl), 1 M, respectively, to yield the desired osmosensitive medium formulation. The medium was further sterilized by exposure to UV light in a biosafety cabinet for 30 min, then cooled, packaged and stored at 4 °C for further use.

1 ml *N*. *glabrata* cells (1×10^6^ c.f.u.·ml^−1^) were co-incubated with 1 ml of 0.25 % HAL-ES (final concentration: 0.125%) at 37 °C for 1 h, followed by aPDT irradiation using a 630-nm wavelength laser at a power density of 60 mW cm^−2^ for 30 min at a distance of 10 cm. Post-irradiation, the cells were centrifuged to remove the supernatant and washed three times with 1 ml of PBS to eliminate residual HAL-ES. The treated and untreated cells were resuspended in PBS and adjusted to a concentration of ~1×10^6^ c.f.u.·ml^−1^. Subsequently, a 10-fold serial dilution was performed across five dilution steps. For each dilution, 5 µl of the *N. glabrata* cell suspension was spotted onto YPD osmosensitive agar plates containing the specified stress-inducing agents. Agar media containing hydrogen peroxide (H_2_O_2_) were used to simulate oxidative stress, SDS was used to simulate membrane perturbation and sodium chloride (NaCl) was used to simulate high osmotic pressure [21,22]. The plates were incubated at 37 °C for 24–48 h.Colony growth on stress-containing plates was compared to standard YPD agar plates (without any stress-sensitive reagents) and to control plates containing only individual stress agents. All experiments were performed in triplicate.

### Antifungal sensitivity assay

The Kirby–Bauer (K-B) disc diffusion method was employed to evaluate the effect of HAL-ES-aPDT on the antifungal susceptibility of fluconazole-resistant *N. glabrata*, following the standardized guidelines recommended by the Clinical and Laboratory Standards Institute for assessing antimicrobial resistance. *N. glabrata* cells (1×10^6^ c.f.u.·ml^−1^) were cultured in YPD medium at 37 °C for 2 h in the presence or absence of HAL-ES-aPDT. The resulting cultures were then inoculated onto Mueller–Hinton agar plates (Hopebio, Qingdao, China) supplemented with 2% glucose and methylene blue (0.5 mg ml^−1^). Fluconazole (25 mg) discs (BKMAM, Hunan, China) were placed at the centre of each plate, followed by incubation at 37 °C for 24 h. Zones of inhibition were measured and recorded. Images of each petri dish were captured using a digital camera (XT2; Fujifilm, Tokyo, Japan). All experiments were performed in triplicate.

### Measurement of rhodamine 6G uptake and glucose-induced efflux

Rhodamine 6G (R6G) uptake and glucose-induced efflux measurement measure the efflux pump activity [23]. *N. glabrata* cells, prepared before and after HAL-ES-aPDT treatment at a concentration of 5×10^7^ c.f.u.·ml^−1^ as previously described, were transferred into YPD broth and incubated at 37 °C for 12 h. Each strain was subsequently subjected to glucose depletion by incubation in a constant-temperature shaker (37 ℃, 200 r.p.m.; BSD-100, Shanghai, China) for 2 h. After centrifugation, the supernatant was discarded, and 1 ml of 10 µM R6G (Nishuo Trading Co., Guangzhou, China) was added. The samples were then co-incubated for 2 h in a CO_2_ incubator under light-protected conditions. After incubation, the bacterial suspensions in Eppendorf tubes were placed on ice and then centrifuged at 12,000 r.p.m. for 1 min at 4 ℃. The supernatant was discarded, and the pellet was washed three times with 1 ml of PBS under the same centrifugation conditions. The washed suspensions were then divided equally into two tubes: one containing 50 µl of *N. glabrata* cell suspension and 950 µl of PBS and the other containing 50 µl of bacterial suspension and 950 µl of PBS supplemented with 2 mM glucose. After 1 min of centrifugation at 12,000 r.p.m. (4 ℃), the fluorescence intensity of R6G in the supernatant was measured at 0.5, 1 and 2 h using a multifunctional microplate reader (excitation, 525 nm; emission, 551 nm). All experiments were performed in triplicate.

### Gene expression analysis by quantitative PCR

For RNA extraction, 2–3 ml of *N. glabrata* cells in the logarithmic growth phase were harvested into 1.5-ml microcentrifuge tubes and centrifuged at 12,000 r.p.m. for 30 s. The supernatant was discarded, and 600 µl of SE buffer (containing 0.1% *β*-mercaptoethanol) was added to resuspend the pellet gently. Subsequently, 100–150 U of lyticase was added, and the mixture was incubated at 37 °C for 15–30 min to digest the cell wall. After centrifugation at 13,000 r.p.m. for 1 min, the supernatant was discarded. The cell pellet was lysed with 350 µl of RLT lysis buffer (containing 1% *β*-mercaptoethanol), mixed thoroughly by pipetting and vortexed vigorously for 20 s. An equal volume of 70% ethanol (prepared with Diethyl pyrocarbonate-treated water) was added, mixed immediately and transferred to an RA adsorption column. The column was centrifuged at 13,000 r.p.m. for 30–60 s, and the flow-through was discarded. To remove protein contaminants, 700 µl of RW1 buffer was added to the column, incubated for 30 s and centrifuged at 12,000 r.p.m. for 30 s. After discarding the flow-through, 500 µl of RW wash buffer was added, and the mixture was centrifuged under the same conditions. This washing step was repeated once. The column was then centrifuged at 13,000 r.p.m. for 2 min to eliminate residual wash buffer. The RA column was transferred to an RNase-free microcentrifuge tube. RNase-free water (30–50 µl), preheated to 70–80 °C, was added to the membrane, incubated at room temperature for 1 min and centrifuged at 12,000 r.p.m. for 1 min. If the expected RNA yield was >30 µg, 30–50 µl of RNase-free water was added, and the step was repeated, combining the eluates.

For cDNA synthesis, the reverse transcription (RT) reaction was assembled on ice using 2 µl of 5×PrimeScript RT Master Mix (Perfect Real Time) (final concentration 1 ×), total RNA and RNase-free dH₂O to a final volume of 10 µl. The reaction volume could be increased as required, with each 10 µl reaction containing up to 500 ng of total RNA. The mixture was gently mixed and incubated at 37 °C for 15 min, followed by enzyme inactivation at 85 °C for 5 s and held at 4 °C.

Quantitative PCR was performed using SYBR^®^ Premix Ex Taq™ II (catalogue no. RR820A; TaKaRa Bio) on a QuantStudio™ 5 real-time PCR system (Thermo Fisher Scientific), as described previously [24,26]. *ACT1* was chosen to be a housekeeping gene [27], and we normalized gene expression by dividing the abundance of each target gene by the geometric mean of the housekeeping gene *ACT1*, and the mean relative expression across three independent experiments was calculated using the 2^–ΔΔCt^ method.

### Candida interactions in the *Galleria mellonella* model

*G. mellonella* was used as the host model for *N. glabrata* infection to evaluate the pathogenicity of HAL-ES-PDT on clinically resistant strains [28]. To establish a suitable *G. mellonella* infection model, larvae ~6 weeks old, 2–3 cm in length and weighing around 300 mg were selected, ensuring comparable vitality levels across groups. Each experimental group consisted of 10 larvae. A microlitre syringe was used to inject 10 µl of fungal suspension into the fourth abdominal segment on the left side of the larvae. Following inoculation, the larvae were placed back into individual petri dishes and incubated at 37 °C. Survival was monitored at 24-h intervals over 120 h.

### Statistical analysis

The statistical significance of the differences between the treatment and control groups was assessed using the paired sample t-test. All results were obtained from three independent experiments, with each value presented as the mean±sd (*n*=3). The significance levels are indicated as follows: ns, no statistical significance; ^∗^*P*<0.05; ^∗∗^*P*<0.01; ^∗∗∗^*P*<0.001 compared to the control group.

## Results

### HAL-ES-aPDT increases the stress sensitivity of *N. glabrata*

**Fig. 1.**
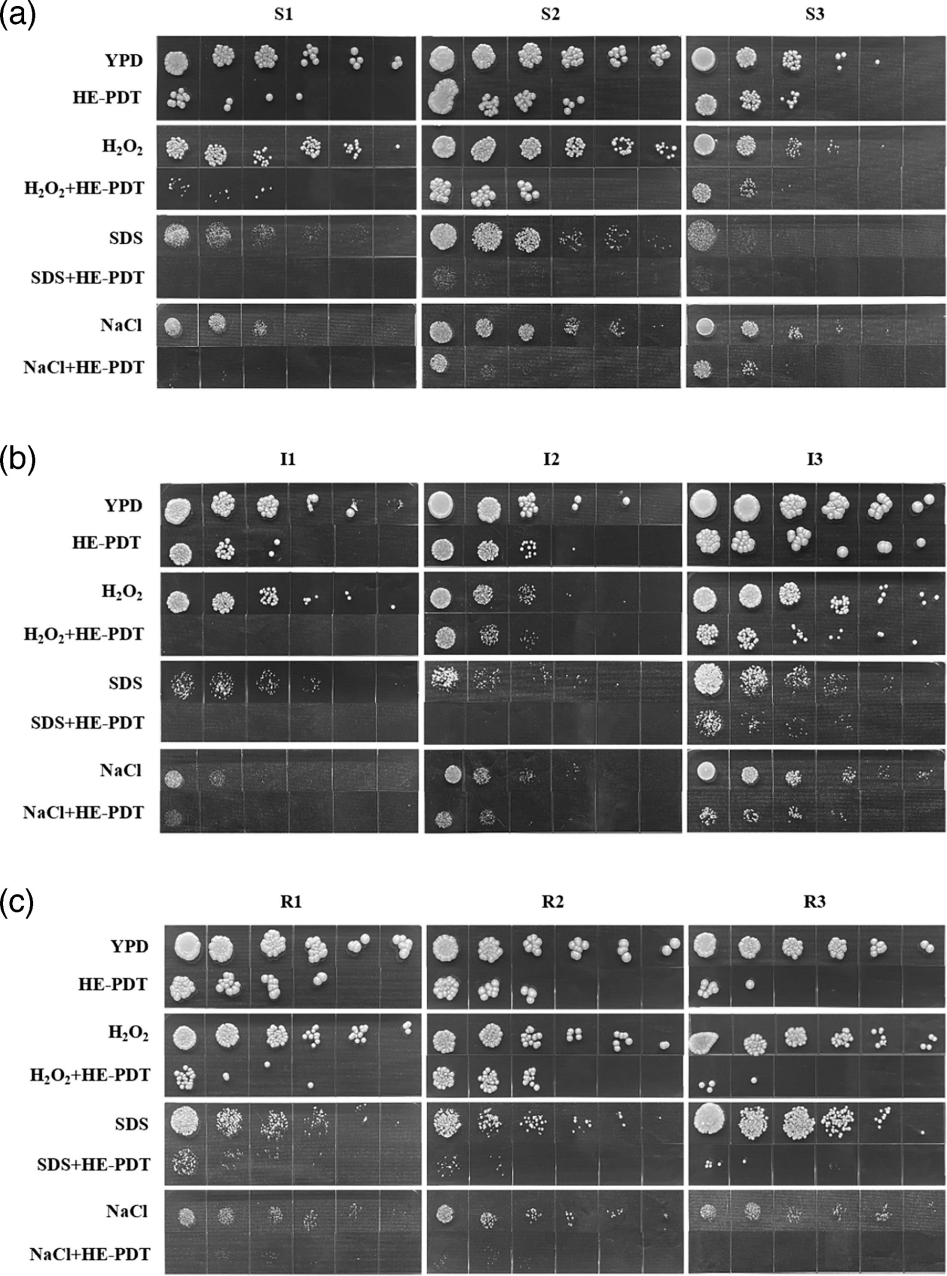
Effect of HAL-ES-aPDT on the stress responses of *N. glabrata*. (a) azole-sensitive, (b) azole-intermediate and (c) azole-resistant.

The impact of HAL-ES-aPDT on the antimicrobial efficacy against drug-resistant *N. glabrata* was assessed using agar media containing hydrogen peroxide (H_2_O_2_), SDS and sodium chloride (NaCl) to simulate oxidative stress, membrane perturbation and high osmotic pressure, respectively. Following HAL-ES-aPDT treatment, the colonies formed by sensitive, intermediate and drug-resistant *N. glabrata* were reduced to varying degrees on each stress-sensitive medium. The colonies exhibited slower growth rates and smaller sizes (Fig. 1). These results demonstrated that HAL-ES-aPDT reduces the tolerance of *N. glabrata* to oxidative stress and hyperosmotic conditions while enhancing the cell membrane’s sensitivity to disruptive agents.

### HAL-ES-aPDT enhances fluconazole susceptibility in drug-resistant *N. glabrata*

**Fig. 2.**
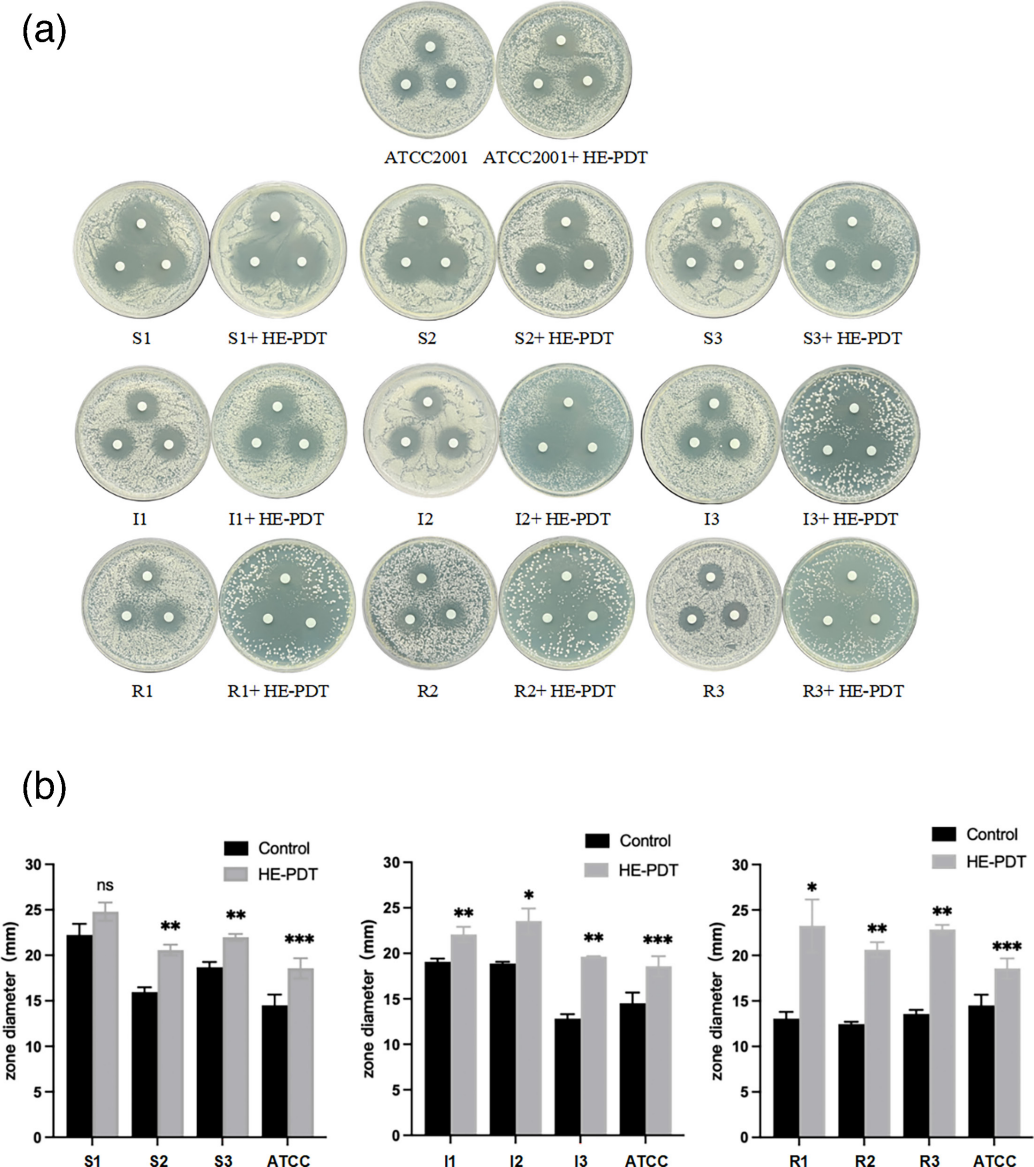
Effect of HAL-ES-aPDT treatment on fluconazole susceptibility. (**a**) Fluconazole inhibitory zone of *N. glabrata*; (**b**) diameter of the fluconazole inhibitory zone of *N. glabrata* (S, sensitive group strain; I, intermediate group strain; R, drug-resistant group strain). The control group was not treated with HAL-ES-aPDT. ns, no statistical significance; *P<0.05; **P<0.01; ***P<0.001, compared with the control group.

The K-B disc diffusion method, recognized for its reproducibility and straightforward interpretation, was employed to evaluate changes in fluconazole susceptibility in *N. glabrata* strains [29]. According to standard criteria, fluconazole resistance is indicated by an inhibitory zone diameter of ≤14 mm, intermediate susceptibility by a diameter between >14 and <19 mm and full susceptibility by a diameter of ≥19 mm [29]. Following HAL-ES-aPDT treatment, the diameters of the inhibition zones of sensitive, intermediate and resistant *N. glabrata* strains increased significantly (*P*<0.05), indicating enhanced fluconazole susceptibility. Notably, the inhibition zone diameters of the drug-resistant strains increased to within the range observed for susceptible strains, suggesting that HAL-ES-aPDT reversed fluconazole resistance and restored antibacterial efficacy (Fig. 2, Table 1).

**Table 1.** Fluconazole inhibitory zone diameter of *N. glabrata* measured using the K-B disc diffusion method (mean±sd, *n*=3)

Strain	Control group (mm)	HAL-ES-aPDT group (mm)	*P*-value
S1	22.7433±0.36350	25.3700±0.50478	0.026
S2	20.5700±0.54065	22.6467±0.35572	0.035
S3	19.2433±0.29872	21.9833±0.37554	0.008
I1	15.9667±0.52918	20.5800±0.59025	0.006
I2	18.8867±0.18610	23.5400±1.38849	0.022
I3	12.8133±0.49319	19.6333±0.05686	0.002
R1	12.0667±0.73330	23.2567±2.89832	0.016
R2	11.4600±0.25239	20.6333±0.82033	0.004
R3	12.5700±0.44396	22.8533±0.50738	0.003
ATCC	14.5133±1.18429	18.5667±1.10970	0.000

### HAL-ES-aPDT reduces drug efflux in drug-resistant *N. glabrata*

**Fig. 3.**
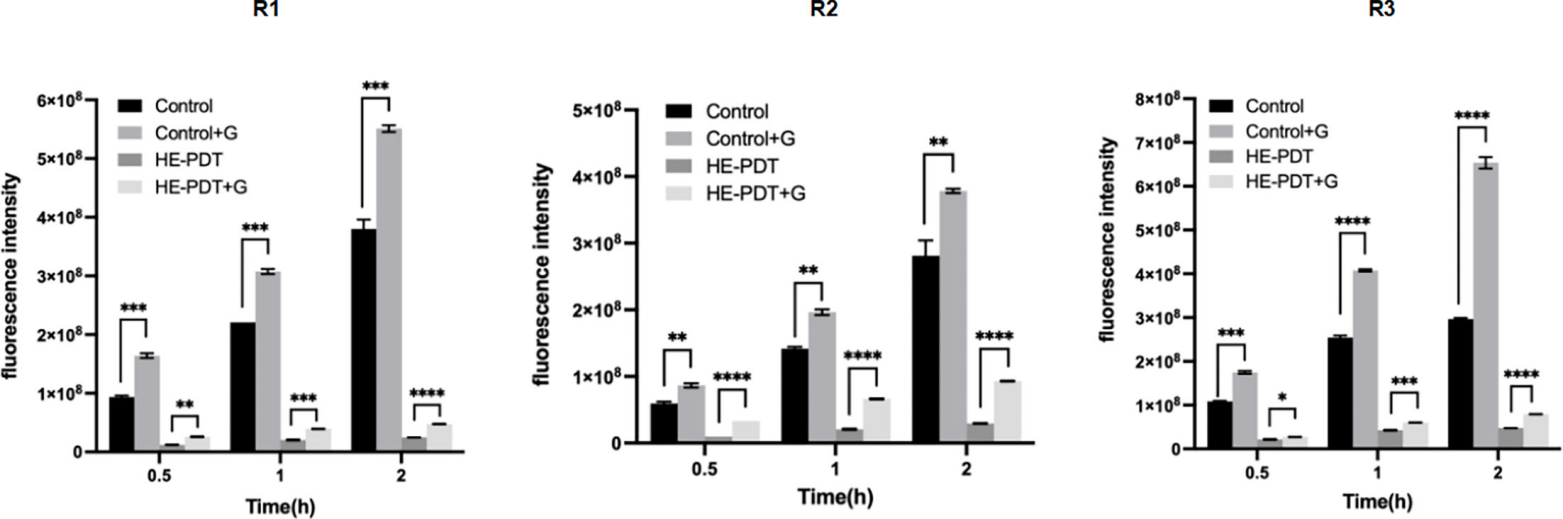
Efflux pump activity in drug-resistant *N. glabrata* before and after HAL-ES-aPDT treatment was assessed by measuring fluorescence intensity. The fluorescence intensity indicates the amount of R6G transported out of the cells in the presence or absence of glucose. ns, no statistical significance; *P<0.05; **P<0.01; ***P<0.001; ****P<0.0001, compared with the control group.

Efflux pump-mediated drug extrusion is a major mechanism underlying azole resistance in *N. glabrata* [30]. R6G was used as a fluorescent substrate to assess efflux pump activity by measuring its fluorescence intensity in the supernatant. Over time (0.5, 1 and 2 h), fluorescence intensity in the control groups progressively increased (Fig. 3), indicating active drug efflux in resistant *N. glabrata*. Upon glucose supplementation, efflux activity increased significantly (*P*<0.05), confirming the involvement of energy-dependent efflux pumps. Following HAL-ES-aPDT treatment, efflux pump activity was significantly attenuated (*P*<0.05). Although glucose addition enhanced efflux activity post-treatment, the magnitude of the increase was substantially lower compared to the untreated control (*P*<0.05). These findings indicated that HAL-ES-aPDT reduces drug efflux in drug-resistant *N. glabrata*, predominantly through the inhibition of energy-consuming efflux mechanisms.

### HAL-ES-aPDT downregulates the expression of genes associated with virulence and drug resistance in *N. glabrata*

**Fig. 4.**
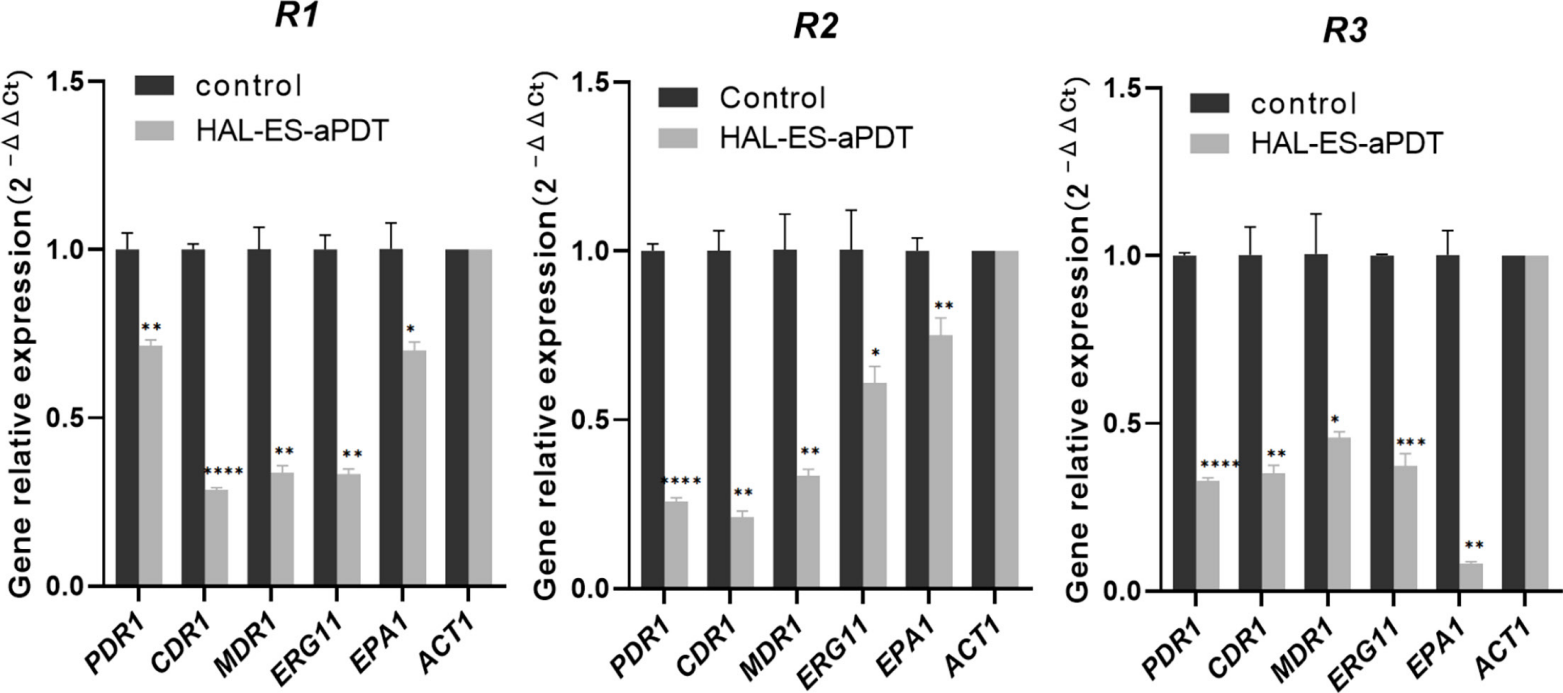
Relative expression levels of genes associated with azole resistance in the control and HAL-ES-aPDT groups were measured by using quantitative real-time PCR (housekeeping gene *ACT1*). Gene expression levels in the control group were normalized to 1, with the control group not receiving HAL-ES-aPDT treatment. *P<0.05; **P<0.01; ***P<0.001, compared with the control group.

CDR1 is an energy-dependent active efflux pump, transcriptionally regulated by PDR1, a key factor contributing to azole resistance in *N. glabrata* [31]. However, *MDR1* functions as a proton motive force-dependent transporter that facilitates passive drug efflux along the electrochemical gradient [32]. Quantitative PCR was used to assess the transcriptional changes of representative genes associated with drug resistance and virulence before and after HAL-ES-aPDT treatment. The analysed genes included the regulatory and structural efflux pump genes *PDR1*, *CDR1* and *MDR1*, the fluconazole target enzyme gene *ERG11* and the epithelial adhesin gene *EPA1*. Following HAL-ES-aPDT treatment, the expression of all tested genes was significantly downregulated (*P*<0.05, Fig. 4). These results indicated that HAL-ES-aPDT reverses azole resistance in *N. glabrata* by downregulating the expression of genes encoding efflux pumps and their regulators, as well as the azole target enzymes. Furthermore, the observed downregulation of *EPA1* indicated a reduction in virulence-related gene expression.

### HAL-ES-aPDT reduces the *in vivo* virulence of clinical *N. glabrata* isolates

**Fig. 5.**
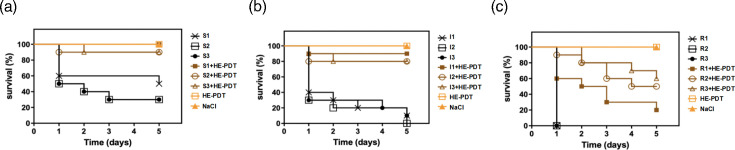
Therapeutic effects of HAL-ES-aPDT treatment on *G. mellonella* larvae infected with (**a**) azole-resistant, (**b**) azole-intermediate and (**c**) azole-sensitive *N. glabrata*, as determined by survival analysis.

*G. mellonella* serves as a widely accepted *in vivo* model for studying *Candida* infections due to its innate immune response, which closely resembles that of mammals, making it suitable for the evaluation of antifungal agents [33,34]. In this study, changes in *N. glabrata* pathogenicity following HAL-ES-aPDT treatment were evaluated through survival analysis using the *G. mellonella*model. In the sensitive and intermediate groups, the 5-day survival rate of larvae infected with *N. glabrata* ranged from 0% to 50%. HAL-ES-aPDT treatment significantly increased survival rates to 80%–100% (*P*<0.05, Fig. 5a and b). In the drug-resistant group, the 1-day post-infection survival rate was 0%. HAL-ES-aPDT elevated the 1-day survival rate to 60%–100% (*P*<0.05), while the 5-day survival rate remained at 20%–60% (*P*<0.05, Fig. 5c). These results indicate that HAL-ES-aPDT significantly reduced the *in vivo* virulence of *N. glabrata* across sensitive, intermediate and drug-resistant strains.

## Discussion

Fungal drug resistance is becoming increasingly prevalent, necessitating the development of novel therapeutic strategies. aPDT has emerged as a promising method for the treatment of pathogenic infections [10]. Unlike conventional antimicrobial drugs that act on specific molecular targets, aPDT induces non-specific oxidative damage to critical biomacromolecules, such as proteins, nucleic acids and cell membranes, through the intracellular generation of ROS [35]. The novel photosensitizer, HAL-ES, has demonstrated high efficiency in mediating photodynamic antimicrobial activity. Previous studies have reported that HAL-ES-aPDT exerts anti-biofilm effects, reverses antifungal resistance and reduces pathogenicity in *C. albicans* [15,16]. Given that *N. glabrata* and *C. albicans* belong to distinct evolutionary branches of fungi [36], this study investigated the therapeutic potential of HAL-ES-aPDT against drug-resistant *N. glabrata*.

Since *N. glabrata* rarely forms robust biofilms and its azole resistance stems mainly from intrinsic efflux-pump up-regulation rather than biofilm-specific factors, whereas even when sparse biofilms do arise the extracellular matrix is limited and efflux-pump overexpression remains dominant, we focused our studies on efflux-pump alterations at the planktonic level [37]. The results demonstrated that HAL-ES-aPDT enhances the susceptibility of *N. glabrata* to environmental stressors, thereby diminishing its survival under conditions of oxidative stress, membrane perturbation and high osmotic pressure. Notably, the treatment acted like a complex, multi-pronged attack, delivering multiple stressors that did not induce cross-protection against these stressors, which is advantageous for limiting adaptive resistance and reducing the likelihood of resistant mutations of *N. glabrata*. HAL-ES-aPDT effectively reversed azole resistance in *N. glabrata*, as confirmed across multiple clinical isolates of resistant strains. When using disc diffusion to evaluate HAL-ES-aPDT-mediated reversal of resistance, we observed that zones near the breakpoint frequently yield false-resistant results due to insufficient assay standardization; consequently, we routinely rely on microdilution MICs for definitive susceptibility determination [38]. HAL-ES-aPDT has been shown to reverse azole resistance in *C. albicans*. However, in contrast to *N. glabrata*, *C. albicans* primarily develops azole resistance through biofilm formation, an acquired resistance mechanism. *N. glabrata*, which exhibits a lower propensity for biofilm formation, exhibits intrinsic azole resistance largely attributed to the overexpression of efflux pumps [3,7]. Further investigations revealed that HAL-ES-aPDT inhibited the activity of both energy-dependent and energy-independent efflux pumps in *N. glabrata*. The efflux pump genes *CDR1* and *MDR1* [39]*,* as well as their upstream transcription regulator *PDR1* [8,31], were significantly downregulated post-treatment. The decrease in efflux activity may correlate both linearly with changes in gene expression and post-transcriptional regulation at play. Previous studies have similarly reported that aPDT inhibits drug efflux in bacteria such as methicillin-resistant *S. aureus* [40] and *Acinetobacter baumannii* [41], likely due to ROS-induced structural and functional damage to the bacterial cell membrane, including membrane rupture and reduced selective permeability, which inhibits active efflux systems [42]. Additionally, the expression of *ERG11*, the gene encoding the target enzyme of fluconazole in *N. glabrata*, was significantly downregulated post-treatment. Thus, the reduction in efflux pump activity and inhibition of target enzyme expression are the primary mechanisms by which HAL-ES-aPDT reverses azole resistance in *N. glabrata*. Our findings demonstrate that HAL-ES-aPDT holds clear potential for combination therapy with fluconazole against azole-resistant infections, even though the present study did not conduct extended follow-up of MIC dynamics beyond a few days post-treatment. Future time-course experiments will be required to establish durability. Future time-course experiments will be required to establish durability.

The epithelial adhesin *EPA1* [43] plays a crucial role in the virulence of *C. glabrata* by enhancing adhesion to host epithelial cells. Its expression is regulated by the upstream transcription factor *PDR1* [43]. In this study, HAL-ES-aPDT treatment significantly downregulated the expression of *EPA1* and *PDR1* in *N. glabrata*, resulting in increased survival rates of *G. mellonella* infected with sensitive, intermediate and drug-resistant *N. glabrata* strains. These findings indicate that HAL-ES-aPDT attenuates the virulence of *N. glabrata*, consistent with previously observed effects in *C. albicans*. This similarity may be attributed to identical structural and metabolic characteristics, including mechanisms of tissue adhesion and invasion [3], and the non-selective damage to cellular components induced by ROS generated during aPDT [35].

Fungal infections are often recurrent and require prolonged antifungal treatment, which can lead to severe adverse effects and the development of drug resistance. Therefore, combining antifungal agents with aPDT presents a promising strategy. This approach may reduce the required drug dosage, limit the emergence of resistant strains and thereby address the challenge of antifungal resistance. HAL and ethosome are clinically safe, yet HAL-ES-aPDT is intended for localized use only, and variables such as tissue penetration and light uniformity limit its applicability to systemic infections. Nowadays, in addition to the classic drug fluconazole, drugs such as voriconazole and posaconazole are utilized in the clinic to fight fungal infections [18,44]. The present study demonstrated that HAL-ES-aPDT enhances the sensitivity of *N. glabrata* to environmental stressors, reverses its intrinsic azole resistance and attenuates its virulence *in vivo*. Due to similarities in the pharmacologic mechanisms of azoles [6], HAL-ES-aPDT will demonstrate comparable or enhanced synergistic effects when combined with voriconazole or posaconazole against azole-resistant. However, resistance mechanisms may vary among fungal species. This investigation focused solely on the effects and mechanisms of HAL-ES-aPDT in fluconazole-resistant *N. glabrata*, and its efficacy against fungal pathogens remains to be determined. Furthermore, as a novel antimicrobial therapy, the clinical application of aPDT requires further optimization, including a selection of photosensitizers and appropriate light dosages tailored to individual cases [45].

## Supplementary material

10.1099/jmm.0.002060 Uncited Table S1.

## Abbreviations

aPDT: antimicrobial photodynamic therapy
HAL-ES-aPDT: hexyl-aminolevulinate ethosome-mediated antimicrobial photodynamic therapy
K-B: Kirby–Bauer
R6G: rhodamine 6G
ROS: reactive oxygen species
RT: reverse transcription
YPD: yeast extract peptone dextrose
