# Supplementary material for: Role and mechanism of hexyl-aminolevulinate ethosome-mediated antimicrobial photodynamic therapy in reversing fluconazole resistance in Nakaseomyces glabrata (Candida glabrata)
Source: J Med Microbiol. 2025 Sep 12;74(9):002060. doi: 10.1099/jmm.0.002060 (PMC12451749; doi:10.1099/jmm.0.002060)
Supplement: Uncited Table S1. [file jmm-74-02060-s001.pdf]

Supplementary Material

Table S1

Sources of the clinical *Nakaseomyces glabrata* isolates

Isolate	Drug sensitivity	Gender	Age	Protopathy	Source of specimens
R1	Resistant	Male	75	Interstitial pneumonia	Aspiration of sputum
R2	Resistant	Male	88	Gallstone	Feces
R3	Resistant	Female	28	Acute lymphocytic leukemia	Feces
I1	Intermediate	Female	43	Chronic nephritis	Midstream urine
I2	Intermediate	Female	32	Sclerosing iliitis	Midstream urine
I3	Intermediate	Male	8	Multiple burns	Secretions
S1	Sensitive	Male	34	Acute lymphocytic leukemia	Feces
S2	Sensitive	Male	20	Multiple intracranial glioblastomas	Spontaneous sputum
S3	Sensitive	Male	29	Multiple burns	Bronchoalveolar lavage fluid
